# The value of preoperative controlling nutritional status score in evaluating short-term and long-term outcomes of patients with colorectal cancer following surgical resection

**DOI:** 10.7150/jca.49383

**Published:** 2020-10-17

**Authors:** Hailun Xie, Chao Nong, Guanghui Yuan, Shizhen Huang, Jiaan Kuang, Ling Yan, Guotian Ruan, Shuangyi Tang, Jialiang Gan

**Affiliations:** 1 Department of Colorectal and Anal Surgery, The First Affiliated Hospital, Guangxi Medical University, Nanning, Guangxi, P.R. China.; 2 Department of Pharmacy, The First Affiliated Hospital, Guangxi Medical University, Nanning, Guangxi, P.R. China.

**Keywords:** colorectal cancer, controlling nutritional status, prognosis, postoperative comorbidities

## Abstract

**Background:** This study aimed to explore the value of controlling nutritional status (CONUT) score in assessing short-term and long-term outcomes of colorectal cancer (CRC) patients, and construct CONUT-based nomograms to predict risk of postoperative comorbidities and survival.

**Methods:** We retrospectively enrolled 512 patients from 2012 to 2014. Patients were categorized into low-CONUT and high-CONUT groups. Logistic regression analysis was used to determine characteristics influencing postoperative comorbidities. Kaplan-Meier survival curve and Cox proportional hazards analysis were used to determine characteristics affecting prognosis. The receiver operating characteristic was used to compare ability of the CONUT score with other immune-nutritional indicators to predict prognosis.

**Results:** Logistic regression analysis suggested that high CONUT score was an independent risk factor affecting postoperative comorbidities (odds ratio, 1.792; 95% confidence interval [CI], 1.113-2.886; P = 0.016). Patients with low-CONUT score had longer disease-free survival (DFS) (P < 0.001) and overall survival (OS) (P < 0.001) compared to those with high-CONUT score, especially at the early stage. CONUT score was an independent factor affecting both DFS (hazard ratio [HR], 1.820; 95% CI, 1.204-2.752; P = 0.005) and OS (HR, 1.815; 95% CI, 1.180-2.792; P = 0.007). The area under the curve of CONUT score was higher than for other immune-nutritional indicators. The CONUT-based nomograms had good predictive capability.

**Conclusions:** CONUT score is a strong independent predictor of postoperative comorbidities and long-term outcomes in CRC patients, and might be a better prognostic factor than other immune-nutritional indicators. The CONUT-based nomograms are conducive to the individualized formulation of follow-up strategies and treatment plans.

## Introduction

Colorectal cancer (CRC) is one of the most common malignancies worldwide. Around 1.8 million people suffer from CRC and 881,000 patients die annually 1. According to the American cancer epidemiological statistics, CRC is the third most common malignant tumor and third leading cause of cancer-related mortality 2. Surgical resection with regional lymph node dissection is still the main treatment for CRC. Despite the continuous development of surgical methods, preoperative and postoperative adjuvant chemoradiotherapy, and the emergence of new molecular targeted drugs, many patients with R0 resection still relapse. The 5- and 10-year survival rates of CRC patients with surgical resection can reach 65%, but the survival rate of patients with recurrence after surgery can be reduced to 10% 3. At present, the tumor-node-metastasis (TNM) staging system is the most useful tool for evaluating the prognosis and guiding treatment options for CRC patients 4. However, the TNM stage has certain limitations because it only focuses on cancer-related characters and cannot fully explain the prognostic heterogeneity of CRC. It is reported that patients with the same TNM stage can still have different outcomes, which indicates that other effective and easily available indicators are urgently needed for individualized postoperative follow-up and treatment stratification.

In recent years, extensive research has supported the critical role of malnutrition and immunological status in cancer advancement and clinical outcomes 5, 6. Host status is not only associated with short-term outcomes such as comorbidities and length of hospital stay, but also with long-term outcomes of patients after treatment. Many immune-nutritional indicators, such as prognostic nutrition indicator (PNI) 7, neutrophil/lymphocyte ratio (NLR) 8, and platelet/lymphocyte ratio (PLR) 9, have been reported as useful prognostic indicators in CRC patients. Recently, controlling nutritional status (CONUT) score 10, a newly proposed immune-nutritional related index, has been reported to be closely related to the prognosis of various tumors including gastric cancer 11, CRC 12, esophageal cancer 13, upper tract urothelial carcinoma 14, and hepatocellular carcinoma 15. CONUT score comprises serum albumin, total cholesterol concentration and lymphocyte count, which respectively reflect host nutritional status, lipid metabolism and immune function. However, the relationship between CONUT score and postoperative comorbidities is still controversial 16, and the efficacy of CONUT score in predicting prognosis compared with other immune-nutritional indicators (such as PNI, NLR, and PLR) remains to be explored.

Therefore, we sought to investigate the prognostic value of the CONUT score in CRC patients, and compare the predictive prognostic efficacy of the CONUT score with other immune-nutritional indicators. We also tried to establish CONUT-based nomograms to individually predict the risk of comorbidities and long-term outcomes in CRC patients after surgery.

## Materials and Methods

### Study population

We retrospectively enrolled 512 CRC patients who underwent surgical resection between January 2012 and December 2014. The inclusion criteria were: (1) patients with pathologically diagnosed CRC; (2) radical resection and negative tumor margin; (3) survival during perioperative period; and (4) complete clinical data and postoperative follow-up. We retrospectively analyzed the following parameters of patients by checking their medical records. Basic patient information included gender, age, body mass index (BMI) (low, <18.5 kg/m^2^; normal, 18.5-24 kg/m^2^; high, ≥ 24 kg/m^2^), and American Society of Anesthesiologists (ASA) grade. Basic tumor information included: tumor location, maximum tumor size, perineural invasion, vascular invasion, histological type, macroscopic type, pathological tumor stage (pT stage), pathological node stage (pN stage), and TNM stage. Basic information of preoperative serology included: neutrophils, lymphocytes, albumin, cholesterol concentration and serum carcinoembryonic antigen (CEA) level (normal, < 5 ng/ml; high, ≥ 5 ng/ml).

### Calculation of CONUT score and other parameters

The CONUT score was calculated based on preoperative albumin concentration, total cholesterol concentration and lymphocyte counts (Table 1). Postoperative comorbidities were classified according to Clavien-Dindo classification 17. The following formulas were used to calculate other immune-nutritional indicators: PNI = albumin concentration (g/L) + 5×lymphocyte count (10^9^/L); NLR = neutrophil/lymphocyte count (10^9^/L); PLR = platelet/lymphocyte count (10^9^/L).

### Postoperative follow-up

Patients underwent routine examinations every 3-4 months within 2 years, and every 6 months within 3 years after surgery, followed by annual outpatient or telephone follow-up visits. Final survival follow-up time was considered the latest follow-up date (September 1, 2019) or death for this study. Disease-free survival (DFS) was defined as the period from the operation to recurrence or metastasis, and overall survival (OS) was defined as the period from the operation to death from all causes.

### Statistical Analysis

Statistical comparisons between the groups were analyzed using the χ^2^ test and student's *t* test. The risk factors of postoperative comorbidities were evaluated using logistic regression analysis. Survival rates were calculated with the Kaplan-Meier method and survival curves were compared using the log-rank test. The independent prognostic factors were assessed by the Cox proportional hazard regression analysis. The predictive prognostic capability of CONUT score was compared with other indicators by the receiver operating characteristic (ROC). The CONUT-based nomograms were constructed based on the results of multivariate analysis. The discriminatory ability of the nomograms was evaluated by consistency index and calibration curve. A *P* value <0.05 was considered statistically significant. All statistical analysis was performed with IBM SPSS version 24.0 and R version 3.5.3.

## Results

### CONUT score grouping and clinicopathological characteristics

**Figure 1:**
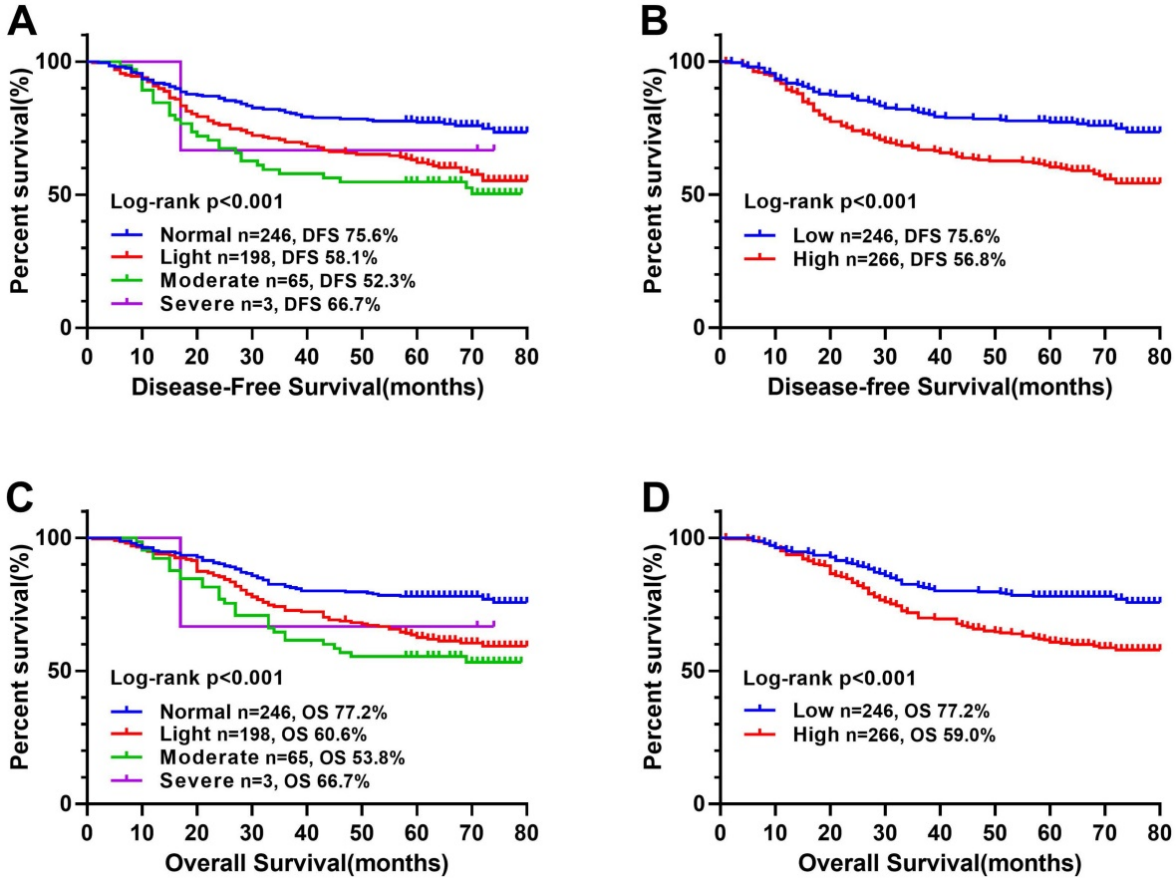
Kaplan-Meier survival curves of CONUT score in CRC patients. **Notes:** A, Disease-free survival curves of normal, light, moderate, and severe group; B, Disease-free survival curves of low- and high-CONUT; C, Overall survival curves of normal, light, moderate, and severe group; D, Overall survival curves of high- and low-CONUT. **Abbreviations:** CONUT: Controlling Nutritional Status; DFS: disease-free survival; OS: overall survival.

**Figure 2:**
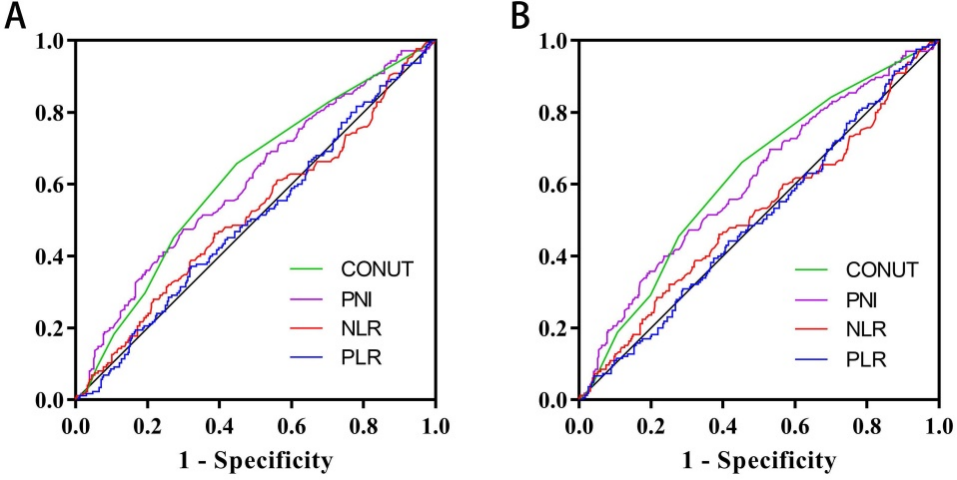
Area under the receiver operating characteristic curves of CONUT score and other immune-nutritional indicators for the prediction of survival. **Notes:** A, Disease-free survival; B, Overall survival. **Abbreviations:** CONUT: Controlling Nutritional Status; PNI: prognostic nutrition indicators; NLR: neutrophil to lymphocyte ratio; PLR: platelet to lymphocyte ratio; DFS: disease-free survival; OS: overall survival.

In a previous study 10, CONUT score was separated into four groups (normal, light, moderate and severe). In this study, we found that there was no significant difference in Kaplan-Meier survival curve among the light, moderate and severe groups, while there was a significant difference between the normal and other groups (Figure 1). In addition, ROC curve analysis for OS revealed that the optimal threshold of CONUT score was 1.5 (sensitivity=0.661, and specificity=0.548) (Figure 2A). According to Kaplan-Meier survival curve and ROC curve, we classified patients more simply into a low-CONUT group and a high-CONUT group, instead of the traditional four classifications of CONUT score. Two hundred and forty-six (48.0%) patients were considered to have a low CONUT score and 266 (52.0%) a high-CONUT score. Ninety-seven (18.9%) patients had recurrence or metastasis, and 165 (32.2%) died. The median follow-up time was 64 months (range 1-80 months).

### Correlation between CONUT score and clinicopathological characteristics

We explored the correlation between CONUT score and clinicopathological characteristics including gender, age, BMI, ASA grade, PNI, NLR, PLR, pT stage, pN stage, TNM stage, tumor size, perineural invasion, vascular invasion, macroscopic type, histological type, CEA, and hospital stays. Patients with high-CONUT were associated with advanced age, low BMI, low PNI, high NLR, high PLR, advanced stage, colon, large tumor size, high CEA, and long hospital stay (Table 2).

### Correlation between CONUT score and postoperative comorbidities

A total of 98 (19.1%) patients had different degrees of postoperative comorbidities, including nine with intestinal obstruction, 12 with gastrointestinal problems, six with anatomic leak, 45 with wound comorbidities, 14 with pulmonary infection, and 12 with other comorbidities. Based on Clavien-Dindo classification, 44 cases were grade I, 39 grade II, ten grade III, four grade IV, and one grade V. The detailed distribution of comorbidities is shown in Table 3. Patients with high CONUT score were more likely to have comorbidities (*p*=0.002), which was mainly grade I (*P* =0.036).

We assessed the risk factors affecting postoperative comorbidities through logistic regression analysis. In univariate analysis, age (*P* = 0.010), CONUT score (*P* = 0.002), surgical approach (*P* = 0.023), and intraoperative blood loss (*P* = 0.001) were associated with postoperative comorbidities. In multivariate analysis, only age (odds ratio [OR], 1.740; 95% confidence interval [CI], 1.088-2.783; *P* = 0.021), CONUT score (OR, 1.792; 95% CI, 1.113-2.886; *P* = 0.016), and intraoperative blood loss (OR, 2.246; 95% CI, 1.403-3.594; *P* = 0.001) were independent factors affecting postoperative comorbidities (Table 4).

### Correlation between CONUT score and survival outcomes

**Figure 3:**
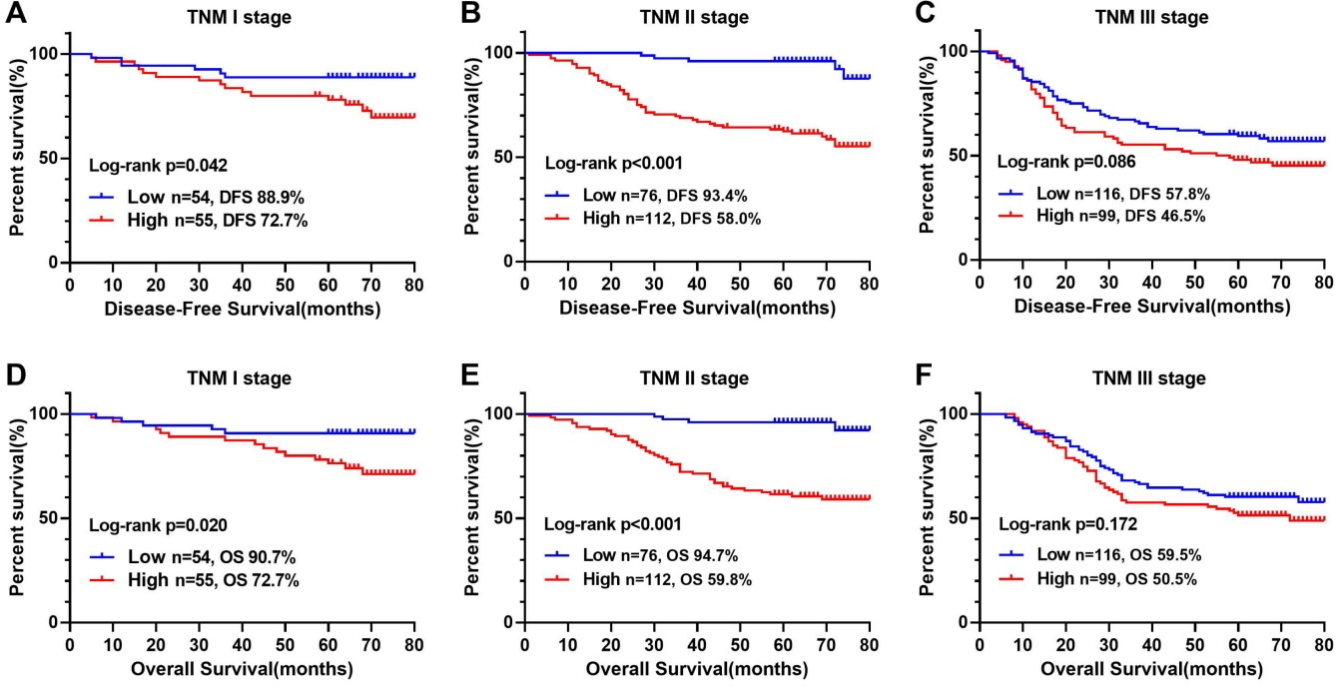
Kaplan-Meier survival curves of low- and high-COUNT groups of CRC patients based on different TNM stages. **Notes:** A, Disease-free survival curves of COUNT score in TNM I; B, Disease-free survival curves of COUNT score in TNM II; C, Disease-free survival curves of COUNT score in TNM III; D, Overall survival curves of COUNT score in TNM I; E, Overall survival curves of COUNT score in TNM II; F, Overall survival curves of COUNT score in TNM III. **Abbreviations:** CRC: colorectal cancer; CONUT: Controlling Nutritional Status; DFS: disease-free survival; OS: overall survival.

The Kaplan-Meier survival curve showed that patients with high CONUT score were significantly lower than those with low CONUT score in terms of both DFS (56.8% vs 75.6%, *P* < 0.001) and OS (59.0% vs 77.2%, *P* < 0.001) (Figure 1B, D). In order to explore further the relationship between CONUT score and TNM stage, we performed a stratified survival analysis based on each TNM stage, and found that CONUT score could distinguish patients with poor prognosis in TNM stage I and II, while among stage III patients, the survival curve had a tendency to distinguish poor prognosis, but there was no significant difference (Figure 3).

**Figure 4:**
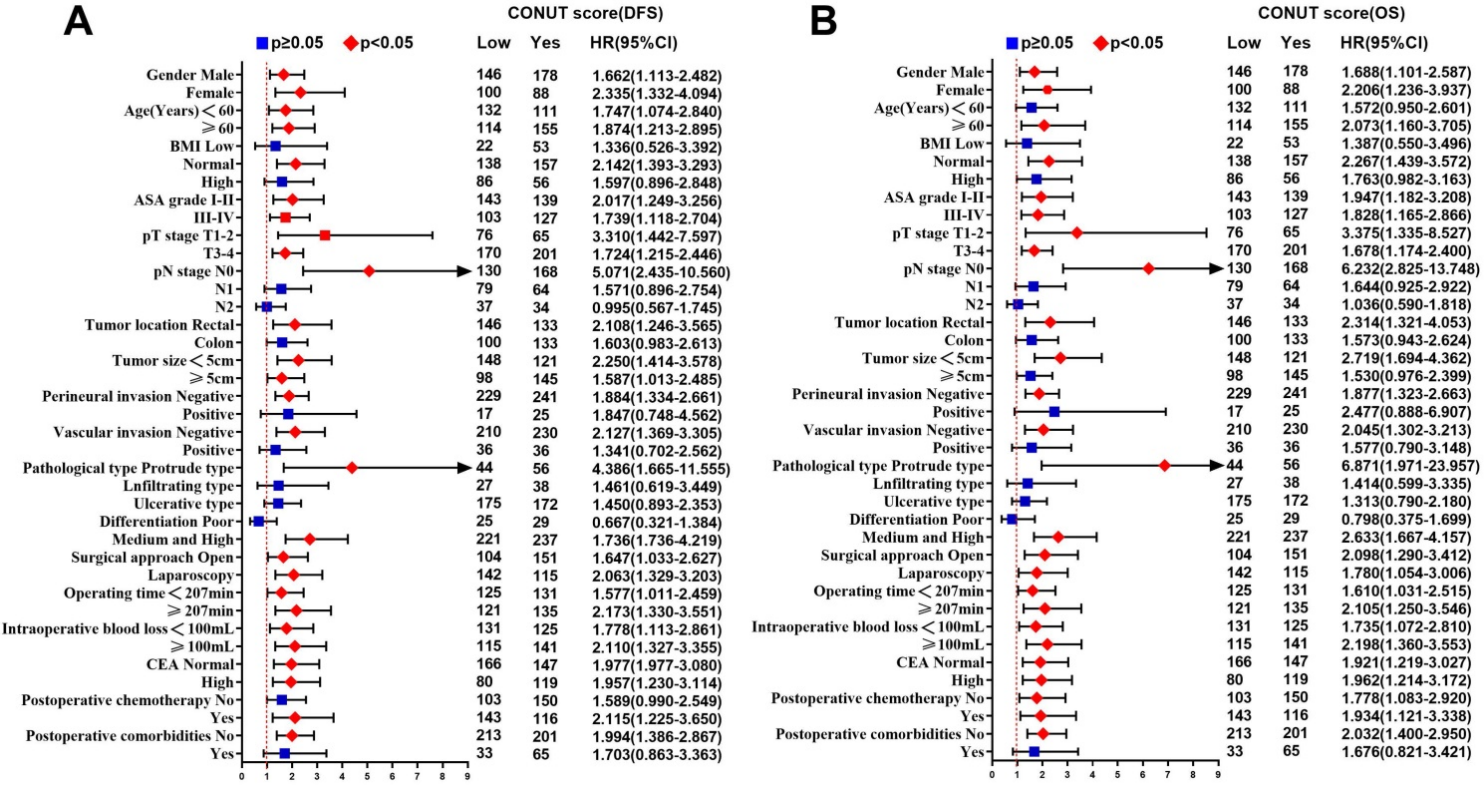
Subgroup multivariate survival analysis of CONUT score in CRC patients. **Notes:** A, subgroup multivariate disease-free survival analysis; B, subgroup multivariate overall survival analysis. **Abbreviations:** CONUT: Controlling Nutritional Status; DFS: disease-free survival; OS: overall survival.

In univariate analysis, ASA grade, CONUT score, PNI, pT3-4 stages, pN stage, perineural invasion, vascular invasion, histological type, CEA, and postoperative comorbidities were related to DFS. Age, ASA grade, CONUT score, PNI, pT3-4 stages, pN stage, perineural invasion, vascular invasion, histological type, CEA, and postoperative comorbidities were related to OS. After adjusting for confounding factors, multivariate survival analysis revealed that only CONUT score, pN stage, CEA, and postoperative comorbidities were independent factors affecting DFS, while only age, CONUT score, pN stage, and postoperative comorbidities were independent factors affecting OS (Table 5). In subgroup multivariate analysis, we found that high CONUT score was an independent risk factor affecting prognosis in most subgroups, regardless of DFS or OS (Figure 4A, 4B).

### Comparison of CONUT score and other parameters in predicting prognosis

**Figure 5:**
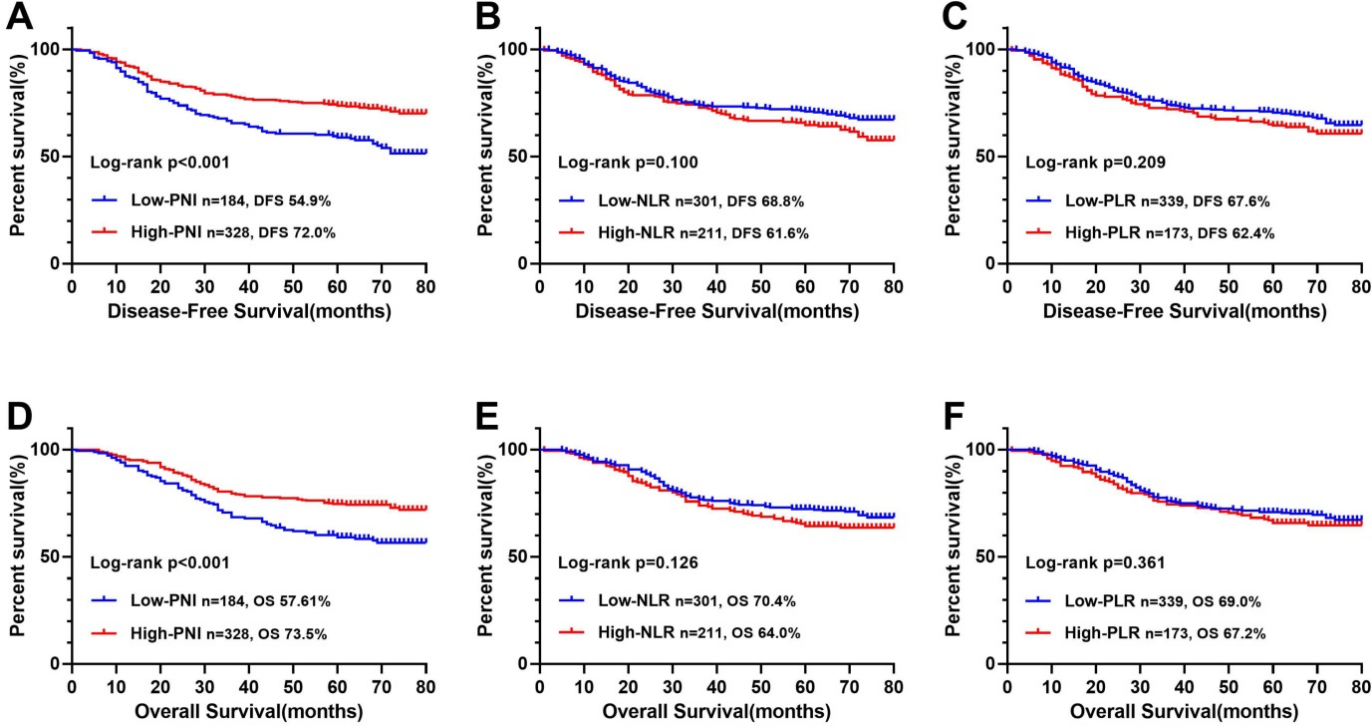
Kaplan-Meier survival curves of immune-nutritional indicators in CRC patients. **Notes:** A, Disease-free survival curves of PNI; B, Disease-free survival curves of NLR; C, Disease-free survival curves of PLR; D, Overall survival curves of PNI; E, Overall survival curves of NLR; F, Overall survival curves of PLR. **Abbreviations:** CONUT: Controlling Nutritional Status; PNI: prognostic nutrition indicators; NLR: neutrophil to lymphocyte ratio; PLR: platelet to lymphocyte ratio; DFS: disease-free survival; OS: overall survival.

The ROC curve determined that the optimal threshold of PNI, NLR, and PLR were 43.88 (sensitivity = 0.473, and specificity = 0.695), 2.41 (sensitivity = 0.461, and specificity = 0.611), and 164.95 (sensitivity = 0.636, and specificity = 0.326), respectively (Figure 2A). Patients were separated to two groups based on these thresholds. Kaplan-Meier analysis of DFS and OS for other immune-nutritional indicators between the two groups was compared (Figure 5). Patients with low PNI had a significantly poorer DFS (*P* < 0.001) and OS (*P* < 0.001) than those with high PNI. However, there was no significant difference between the two groups in NLR (DFS, *P* = 0.100; OS, *P* = 0.126), and PLR (DFS, *P* = 0.209; OS, *P* = 0.361). We compared the predictive prognostic abilities of CONUT score with other immune-nutritional indicators by ROC curve (Figure 2). Comparison of the area under the curve (AUC) values indicated the predictive ability of CONUT score (DFS, 0.617, 95% CI: 0.566-0.668, *P*<0.001; OS, 0.620, 95% CI: 0.569-0.671, *P*<0.001) was better than that of other immune-nutritional indicators: PNI (DFS, 0.607, 95% CI: 0.555-0.659, *P*<0.001; OS, 0.607, 95% CI: 0.555-0.660, *P*<0.001), NLR(DFS, 0.518, 95% CI: 0.464-0.571, *P*=0.511; OS, 0.515, 95% CI: 0.460-0.570, *P*=0.588), PLR (DFS, 0.505, 95% CI: 0.452-0.557, *P*=0.344; OS, 0.501, 95% CI: 0.448-0.554, *P*=0.974).

### Establishing CONUT-based nomograms in predicting outcomes

**Figure 6:**
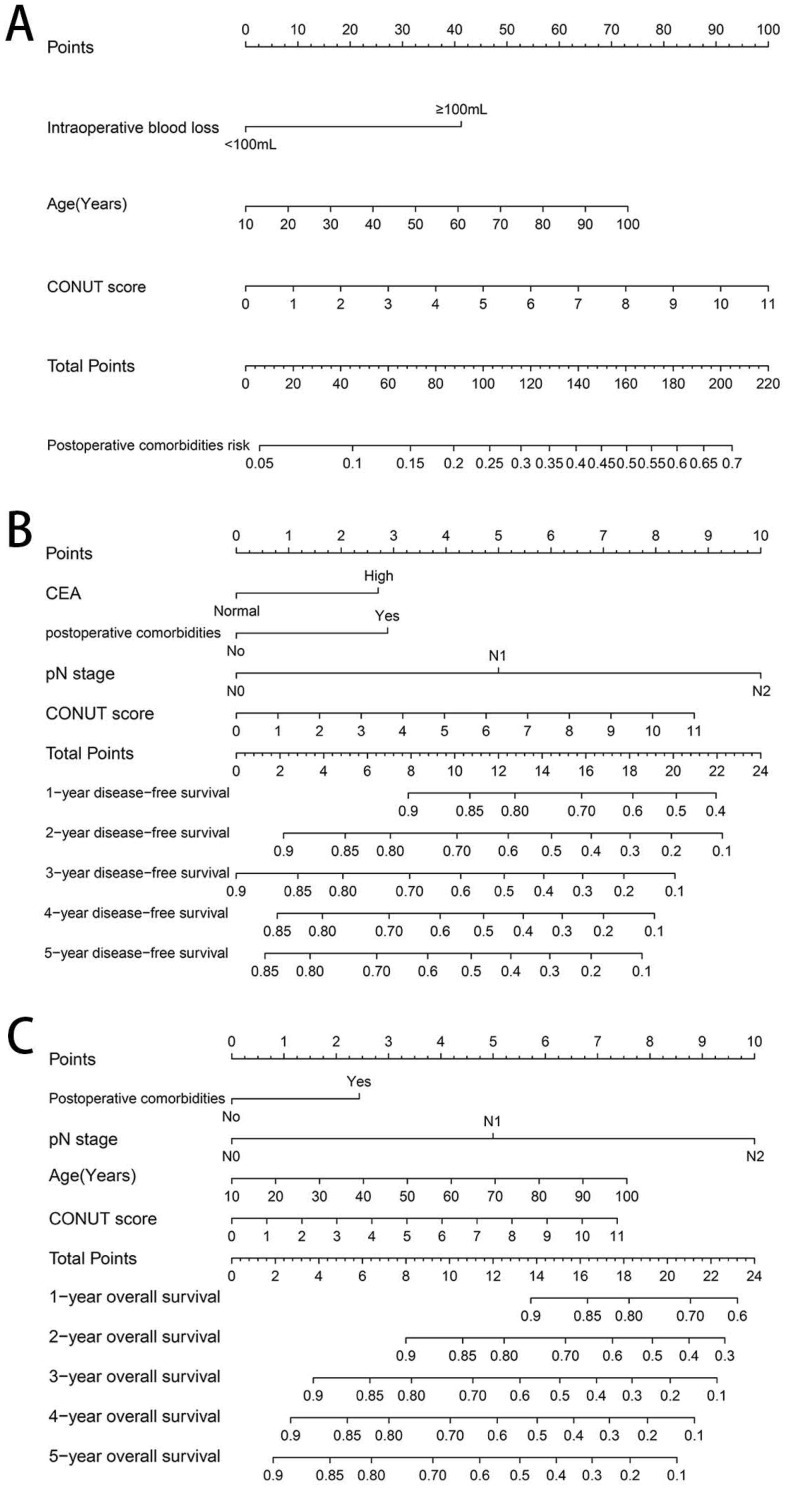
Construction of CONUT-based nomograms in CRC patients. **Notes:** A, CONUT-based nomograms of complication risk; B, CONUT-based nomograms of disease-free survival; C, CONUT-based nomograms of overall survival. **Abbreviations:** CONUT: Controlling Nutritional Status; DFS: disease-free survival; OS: overall survival.

**Figure 7:**
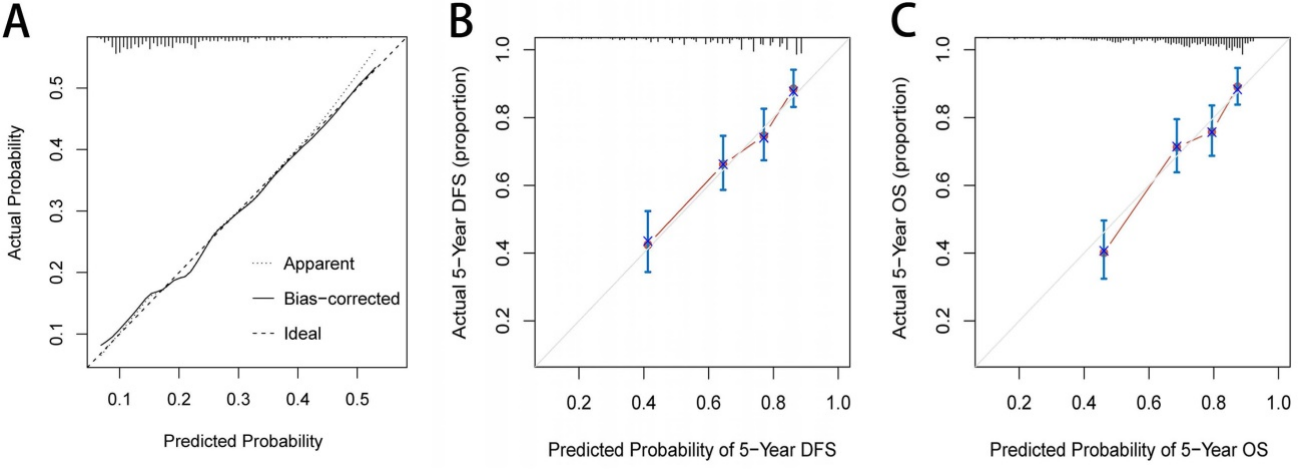
The calibration curves for predicting complication risk (A), disease-free survival (B) and overall survival (C) in CRC patients. **Notes:** The X axis presents the predicted probability and the Y axis shows the actual probability. The calibration lines fit along with the 45 reference.

Based on the three independent factors affecting the comorbidities identified in the logistic regression analysis, a postoperative comorbidities risk nomogram was developed to predict postoperative short-term outcomes. Age and CONUT score were used as continuous variables to improve predictive accuracy. Comorbidities risk increased with intraoperative blood loss, age, and CONUT score (Figure 6A). The C-index for the nomogram was 0.664 (95% CI, 0.602-0.726), and the calibration curves for the probability of comorbidities showed good consistency between the prediction of the nomogram and actual observation (Figure 7A). Based on multivariate survival analysis of DFS, we developed a nomogram, and used the CONUT score as a continuous variable to improve predictive accuracy. The risk of postoperative recurrence and metastasis within 1-5 years increased with preoperative CEA, emergence of postoperative comorbidities, progress of pathological N staging and increase in CONUT score (Figure 6B). Similarly, based on multivariate survival analysis of OS, we developed a nomogram, and used age and CONUT score as continuous variables to improve predictive accuracy. The risk of postoperative mortality within 1-5 years increased with emergence of postoperative comorbidities, progress of pathological N staging, and increased age and CONUT score (Figure 6C). The C-index for DFS and OS nomograms was 0.705 (95% CI, 0.665-0.745) and 0.702 (95% CI, 0.663-0.741), respectively. The calibration curve for probability of postoperative 1-5 years DFS and 1-5 years OS showed consistency between the nomogram prediction and actual observation (Figure 7B, 7C).

**Figure 8:**
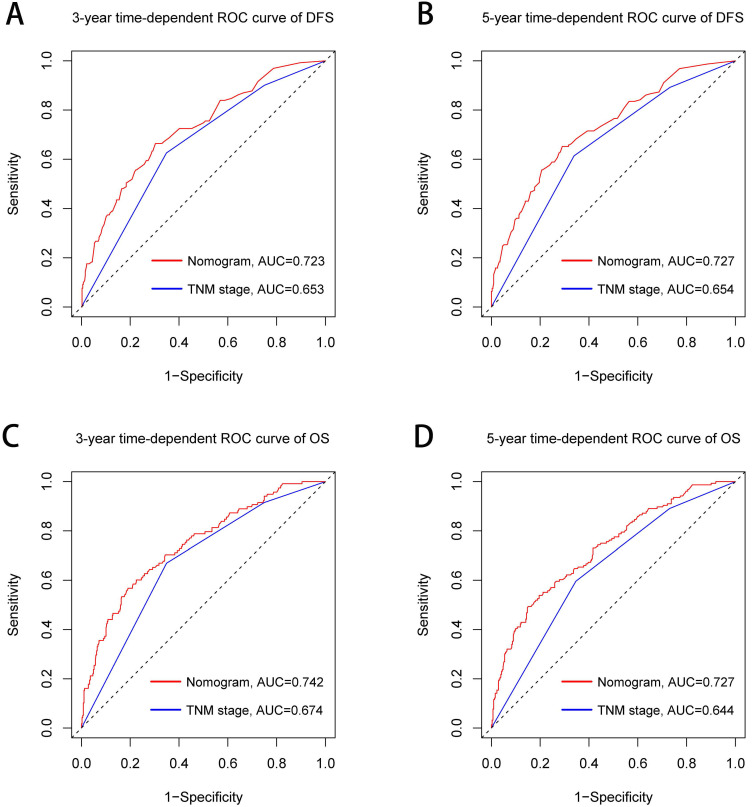
Comparison of the ability of prognostic nomograms and TNM stage for predicting prognosis in CRC patients at 3-year and 5-year point. **Notes:** A, DFS at 3-year point; B, DFS at 5-year point; C, OS at 3-year point; D, OS at 5-year point. **Abbreviations:** DFS: disease-free survival; OS: overall survival.

The TNM stage system is widely recognized as the most effective score system to evaluate the prognosis of CRC patients. We further compared the predictive ability between the prognostic nomograms and the TNM stage system using time-dependent ROC. Compared with the traditional TNM stage system, our prognostic nomograms had better resolution and accuracy in predicting the 3-year and 5-year DFS (3-year AUC: 0.723 vs 0.653; 5-year AUC: 0.727 vs 0.654, Figure 8A, 8B) and OS (3-year AUC: 0.742 vs 0.674; 5-year AUC: 0.727 vs 0.644, Figure 8C, 8D) of CRC patients.

## Discussion

The CONUT score, calculated based on albumin concentration, total cholesterol concentration and total lymphocyte count, is an effective and easy screening tool for evaluating immune-nutritional status. Albumin concentration is one of the most common parameters for assessing nutritional status, and albumin reduction is thought to be related to systemic inflammation affecting catabolism and anabolism of liver cells 18. Hypoalbuminemia also may reduce patients' tolerance to surgery, leading to poorer prognosis 19. Lymphocyte count is an important parameter of immune status, and plays a key role in the host anticancer immunity by inducing cytotoxicity and inhibiting the growth, invasion and migration of tumor cells. The change in its quantity reflects the steady state relationship between tumor progression and tumor resistance 20, 21. Low lymphocyte count is reported as a risk factor for poor prognosis in CRC patients 22, 23. Total cholesterol concentration is thought to be linked with prognosis of patients with various cancers 24, 25. It affects the killing effect of immune cells on cancer cells by affecting the fluidity of cell membranes 26, 27. It is also involved in various cell metabolic pathways, which is considered as an indicator of patients' caloric reserve 28. Because tumor tissue can reduce plasma cholesterol concentration, to some extent, change in cholesterol concentration reflects tumor load and nutritional status. Therefore, the CONUT score, which combines these indicators, is a comprehensive indicator of nutritional status, systemic inflammatory response and immune response.

In our study, high CONUT score was associated with large tumors, advanced tumor stage and colon cancer, suggesting that higher CONUT score was associated with more aggressive tumor phenotypes and disease micrometastatic growth in CRC patients, which might worsen tumor prognosis. In addition, high CONUT score was also associated with advanced age, low BMI and high CEA, indicating that higher CONUT score reflected disease severity in CRC patients. It is worth noting that patients with high CONUT score often have longer hospital stay, which may be because patients with high CONUT score tend to have poorer short-term outcomes, leading to prolonged hospital stay.

Tumor outcomes are affected by tumor behavior, as well as host conditions such as nutrition, inflammation and immunity. Comorbidities may amplify the systemic inflammatory response and accelerate suppression of tumor immunity, leading to tumor progression 29. Approximately 19.1% patients in our study suffered postoperative comorbidities. We found that advanced age, high CONUT score, and increased intraoperative bleeding were independent risk factors for comorbidities. Because the body's aging, the elderly patients were more likely to suffer comorbidities, high-CONUT score reflected the immune nutrition is in a state of disorder and self-healing function in inhibition, and increased intraoperative bleeding further damages the self-healing function of patients. In survival analysis, we found that postoperative comorbidities was an independent risk factor affecting DFS and OS. Previous studies have shown that postoperative comorbidities can affect prognosis of patients, and severity of the comorbidities is related to the survival time of malignancy 30. Systemic inflammation is significantly exacerbated by surgical comorbidities, which is a high-risk factor for tumor growth and spread. Systemic inflammation caused by postoperative comorbidities may have a secondary detrimental effect on survival by increasing the risk of cancer recurrence 31. Preoperative CEA is an independent factor affecting DFS. The increase in preoperative CEA indicates that tumors are relatively advanced and have micrometastases, which increases the risk of postoperative tumor recurrence and metastasis. Age is an independent factor that affects OS, which may be related to the increased risk of non-tumor-related death with age. High CONUT score is an independent risk factor for DFS and OS in CRC patients. In addition, the multivariate subgroup analysis showed that high-CONUT score is a risk factor affecting prognosis in most subgroups. In TNM-based subgroup analysis, although stage III patients with high CONUT score tended to have poor prognosis, it failed to show a significant difference compared with those with low CONUT score. This indicates that CONUT score was associated with survival in patients with early stage CRC, but not in patients with advanced stage disease. It might be that at the early stage of tumors, immune-nutritional factors have a greater impact on patients. Advanced tumors often have greater aggressiveness, such as higher infiltration, larger size, and local micrometastasis, and they may even have preoperative comorbidities such as bleeding, obstruction and perforation. At that time, the invasive nature of the tumor exerts the main influence. In summary, CONUT score could be a useful tool for evaluating short- and long-term outcomes of CRC patients, and assist TNM staging to stratify further CRC patients with poor prognosis. Clinicians should be cautious when considering surgery for malnourished patients with high CONUT score. Before surgery, these patients should strengthen nutritional intervention, and strive to reduce CONUT score to normal levels before planning surgery. During surgery, these patients should consider more extensive resection rather than marginal resection. After surgery, they should undergo closer nutritional monitoring.

In our study, through comparing their AUC values, we confirmed that CONUT score is more accurate in predicting prognosis than other immune-nutritional indicators (PNI, NLR, and PLR) are. In addition, it is worth noting that the CONUT score and PNI have common parameters. The Kaplan-Meier survival curve showed that both the CONUT score and PNI can distinguish patients with poor prognosis, but the CONUT score can more accurately detect patients with poor prognosis than PNI can. This might be due to the greater emphasis on lymphocyte count and additional total cholesterol concentration in CONUT score.

We established a comorbidities nomogram for individualized assessment of the risk of postoperative comorbidities. The C-index and calibration curve performed well, confirming the predictive accuracy of the nomogram. Similarly, we constructed the novel and effective DFS and OS nomograms. The C-index and calibration curve confirmed that these nomograms have good predictive accuracy. Moreover, we compared the ability of prognostic nomograms with the TNM stage in predicting the prognosis of CRC patients. The results showed that our prognostic nomograms were superior to the TNM stage in predicting the 3-year survival or 5-year survival of CRC patients. Through individualized evaluation of CONUT-based nomograms for each patient, we could detect early high-risk populations prone to postoperative comorbidities and poor long-term prognosis, which is conducive to the individualized formulation of early immune-nutritional intervention, postoperative treatment and follow-up plan.

The limitations of the study should be noted. The study was retrospective and included patients from a single institution. Multicenter prospective studies with more patients should be conducted to confirm our findings. In addition, CONUT-based nomograms were formulated based on a limited sample. Although we had performed internal verification to prove that these had moderate predictive accuracy, they still need to be verified with larger external samples in multiple centers. We plan to conduct these useful explorations in the future.

## Conclusion

Our study demonstrated that CONUT score is a strong independent predictor of postoperative comorbidities and long-term outcomes in CRC patients, and might be a more prognostic factor than other immune-nutritional indicators. The CONUT-based nomograms are conducive to the individualized formulation of follow-up strategies and treatment plans for CRC patients, which may bring great benefits to clinical practice.

## Figures and Tables

**Table 1:** Nutritional status assessment according to CONUT scoring system

Parameters	Malnutrition status			
	Normal	Light	Moderate	Severe
Serum albumin (g/dL)	≥3.50	3.00-3.49	2.50-2.99	<2.50
Score	0	2	4	6
Total lymphocyte count	≥1600	1200-1599	800-1199	<800
Score	0	1	2	3
Total cholesterol (mg/dL)	≥180	140-179	100-139	<100
Score	0	1	2	3
Total score	0-1	2-4	5-8	9-12

**Table 2:** The relationships between the CONUT score and clinicopathological factors of CRC patients

Features	Total(n = 512)	CONUT score		*x^2^/t*	*P* value
		Low(n = 246)	High(n = 266)		
**Gender**				3.150	0.076
Male	324 (63.3%)	146 (59.3%)	178 (66.9%)		
Female	188 (36.7%)	100 (40.7%)	88 (33.1%)		
**Age (Years)**				7.294	0.007
<60	243 (47.5%)	132 (53.7%)	111 (41.7%)		
≥60	269 (52.5%)	114 (46.3%)	155 (58.3%)		
Age (Years)	58.48±13.22	56.93±11.33	59.91±14.63	-2.590	0.010
**BMI**				19.624	<0.001
Low	75 (14.6%)	22 (8.9%)	53 (19.9%)		
Normal	295 (57.6%)	138 (56.1%)	157 (59.0%)		
High	142 (27.8%)	86 (35%)	56 (21.1%)		
**ASA grade**				1.783	0.182
I-II	282 (55.1%)	143 (58.1%)	139 (54.0%)		
III-IV	230 (44.9%)	103 (41.9%)	127 (46.0%)		
PNI	45.66±6.15	49.83±4.38	41.80±4.91	19.560	<0.001
NLR	2.77±2.25	1.99±0.78	3.49±2.85	-8.030	<0.001
PLR	158.81±84.03	125.00±41.81	190.09±99.79	-9.489	<0.001
**pT stage**				2.671	0.102
T1-2	141 (27.5%)	76 (30.9%)	65 (24.4%)		
T3-4	371 (72.5%)	170 (69.1%)	201 (75.6%)		
**pN stage**				5.773	0.056
N0	298 (58.2%)	130 (63.1%)	168 (63.1%)		
N1	143 (27.9%)	79 (25.3%)	64 (25.3%)		
N2	71 (13.9%)	37 (11.6%)	34 (11.6%)		
**TNM stage**				7.477	0.024
I stage	109 (21.3%)	54 (22.0%)	55 (20.7%)		
II stage	188 (36.7%)	76 (30.9%)	112 (42.1%)		
III stage	215 (42.0%)	116 (47.2%)	99 (37.2%)		
**Tumor location**				4.505	0.034
Rectal	279 (54.5%)	146 (59.3%)	133 (50.0%)		
Colon	233 (45.5%)	100 (40.7%)	133 (50.0%)		
**Tumor size**				11.036	0.001
< 5 cm	269 (52.5%)	148 (60.2%)	121 (45.5%)		
≥ 5 cm	243 (47.5%)	98 (39.8%)	145 (54.5%)		
**Perineural invasion**				1.051	0.305
Negative	470 (91.8%)	229(93.1%)	241 (90.6%)		
Positive	42 (8.2%)	17 (6.9%)	25 (9.4%)		
**Vascular invasion**				0.128	0.720
Negative	440 (85.9%)	210 (85.4%)	230 (86.5%)		
Positive	72 (14.1%)	36 (14.6%)	36 (13.5%)		
**Macroscopic type**				2.550	0.279
Protrude type	100 (19.5%)	44 (17.9%)	56 (18.2%)		
Infiltrating type	65 (12.7%)	27 (11.0%)	38 (14.1%)		
Ulcerative type	347 (67.8%)	175 (71.1%)	172 (67.7%)		
**Histological type**				0.074	0.785
Poor	54 (10.5%)	25 (10.2%)	29 (10.6%)		
Medium and High	458 (89.5%)	221 (89.8%)	237 (89.4%)		
**CEA**				8.028	0.005
< 5 ng/ml	313 (61.1%)	166 (67.5%)	147 (54.5%)		
≥ 5 ng/ml	199 (38.9%)	80 (32.5%)	119 (45.5%)		
Hospital stays	14.62±7.244	13.80±5.520	15.38±8.476	-2.503	0.013

**Table Note:** CRC: colorectal cancer; BMI: body mass index; ASA grade: Anesthesiologists grade; CONUT score: controlling nutritional status score; PNI: prognostic nutrition indicators; NLR: neutrophil/lymphocyte ratio; PLR: platelet/lymphocyte ratio.

**Table 3:** Details of postoperative comorbidities according to Clavien-Dindo classification

Grade	Total (n=512)	CONUT score			
		Low (n = 246)	High (n = 266)	X^2^	*p*
Total comorbidities	98 (19.1%)	33 (13.4%)	65 (24.4%)	10.031	0.002
Grade I	44 (8.6%)	14 (14.4%)	30 (10.8%)	4.414	0.036
Grade II	39 (8.2%)	14 (5.7%)	25 (9.2%)	2.237	0.135
Grade III	10 (7.6%)	5 (2.0%)	5 (1.9%)	0.019	0.891
Grade IV	4 (0.8%)	0 (0.0%)	4 (1.5%)	3.700	0.054
Grade V	1 (0.2%)	0 (0.0%)	1 (0.4%)	0.927	0.336

**Table 4:** Univariate and multivariate Logistic regression analysis of postoperative comorbidities in CRC patients

Feature	Univariate analysis		Multivariate analysis	
	OR (95%CI)	*p*	OR (95%CI)	*p*
Gender (Female)	0.897 (0.566-1.423)	0.644		
Age (≥60 years)	1.817 (1.152-2.867)	0.010	1.740 (1.088-2.783)	0.021
**BMI**		0.900		
Low	1.000			
Normal	1.167 (0.601-2.266)			
High	1.120 (0.540-2.324)			
ASA grade (III-IV)	0.902 (0.578-1.406)	0.648		
CONUT score (High)	2.087 (1.316-3.310)	0.002	1.792 (1.113-2.886)	0.016
pT stage (T3-4)	1.137 (0.688-1.878)	0.617		
**pN stage**		0.597		
N0	1.000			
N1	0.863 (0.519-1.437)			
N2	0.712 (0.353-1.437)			
Tumor location (Colon)	0.832 (0.533-1.298)	0.417		
Tumor size (≥ 5 cm)	1.193 (0.768-1.853)	0.433		
Perineural invasion (Positive)	1.562 (0.756-3.229)	0.229		
Vascular invasion (Positive)	1.132 (0.611-2.098)	0.694		
**Macroscopic type**		0.261		
Protrude type	1.000			
Infiltrating type	1.251 (0.530-2.957)			
Ulcerative type	1.637 (0.879-3.046)			
Histological type (Medium and High)	0.917 (0.454-1.850)	0.808		
Surgical approach (Open)	1.683 (1.075-2.634)	0.023	1.455 (0.915-2.313)	0.113
Operating time (median, ≥ 207 min)	1.355 (0.870-2.111)	0.179		
Intraoperative blood loss (median, ≥ 100 mL)	2.300 (1.450-3.648)	<0.001	2.246 (1.403-3.594)	0.001
CEA(High)	1.362 (0.872-2.125)	0.174		

**Table Note:** CRC: colorectal cancer; BMI: body mass index; ASA grade: Anesthesiologists grade; CONUT score: controlling nutritional status score.

**Table 5:** Univariate and multivariate survival analysis of clinicopathological characteristics in CRC patients

Feature	Disease-free survival				Overall survival			
	Univariate		Multivariate		Univariate		Multivariate	
	HR (95%CI)	*p*	HR (95%CI)	*p*	HR (95%CI)	*p*	HR (95%CI)	*p*
Gender (Female)	0.768 (0.558-1.057)	0.105			0.760 (0.546-1.057)	0.103		
Age (≥60 years)	1.327 (0.982-1.794)	0.065			1.446 (1.058-1.976)	0.021	1.543 (1.103-2.158)	0.011
**BMI**		0.662				0.981		
Low	1.000				1.000			
Normal	1.213 (0.773-1.904)				1.045 (0.668-1.634)			
High	1.104 (0.669-1.821)				1.044 (0.636-1.714)			
ASA grade (III-IV)	1.807 (1.340-2.437)	<0.001	1.129 (0.808-1.578)	0.476	1.927 (1.415-2.625)	<0.001	1.105 (0.774-1.576)	0.584
CONUT score (High)	1.963 (1.436-2.683)	<0.001	1.847 (1.339-2.548)	<0.001	1.981 (1.435-2.735)	<0.001	1.838 (1.317-2.564)	<0.001
pT stage (T3-4)	2.100 (1.417-3.112)	<0.001	1.380 (0.910-2.094)	0.130	2.173 (1.439-3.284)	<0.001	1.503 (0.966-2.338)	0.071
**pN stage**		<0.001		<0.001		<0.001		<0.001
N0	1.000		1.000		1.000		1.000	
N1	1.624 (1.139-2.316)		1.473 (1.003-2.165)		1.531 (1.057-2.219)		1.440 (0.961-2.156)	
N2	4.563 (3.173-6.561)		3.775 (2.431-5.861)		4.822 (3.334-6.972)		4.373 (2.776-6.889)	
Tumor location (Colon)	0.831 (0.616-1.123)	0.229			0.779 (0.570-1.063)	0.115		
Tumor size (≥ 5 cm)	1.274 (0.947-1.714)	0.110			1.305 (0.961-1.771)	0.088		
Perineural invasion (Positive)	1.860 (1.189-2.910)	0.007	1.260 (0.773-2.055)	0.354	1.679 (1.040-2.709)	0.034	1.157 (0.686-1.951)	0.584
Vascular invasion (Positive)	1.889 (1.314-2.715)	0.001	1.235 (0.819-1.862)	0.315	1.723 (1.176-2.524)	0.005	1.076 (0.695-1.664)	0.743
**Macroscopic type**		0.481				0.499		
Protrude type	1.000				1.000			
Infiltrating type	1.256 (0.726-2.171)				1.346 (0.772-2.348)			
Ulcerative type	1.282 (0.856-1.922)				1.257 (0.826-1.911)			
Histological type (Poor)	2.006 (1.346-2.989)	0.001	1.143 (0.744-1.757)	0.541	1.936 (1.281-2.925)	0.002	1.204 (0.766-1.893)	0.422
Surgical approach (Open)	1.004 (0.746-1.350)	0.981			1.016 (0.749-1.379)	0.919		
Operating time (median, ≥ 207 min)	1.162 (0.863-1.563)	0.323			1.253 (0.922-1.703)	0.150		
Intraoperative blood loss (median, ≥ 100 mL)	1.333 (0.989-1.797)	0.059			1.341 (0.986-1.823)	0.062		
CEA(High)	1.755 (1.305-2.362)	<0.001	1.466 (1.079-1.992)	0.014	1.677 (1.235-2.276)	0.001	1.338 (0.973-1.839)	0.074
Postoperative chemotherapy (Yes)	1.106 (0.822-1.488)	0.505			1.093 (0.806-1.484)	0.567		
Postoperative comorbidities (Yes)	1.591 (1.133-2.233)	0.007	1.576 (1.113-2.231)	0.010	1.514 (1.063-2.155)	0.021	1.480 (1.028-2.131)	0.035

**Table Note:** CRC: colorectal cancer; BMI: body mass index; ASA grade: Anesthesiologists grade; CONUT score: controlling nutritional status score.
